# Supplementary material for: Putative fossil blood cells reinterpreted as diagenetic structures
Source: PeerJ. 2021 Dec 16;9:e12651. doi: 10.7717/peerj.12651 (PMC8684720; doi:10.7717/peerj.12651)
Supplement: Supplemental Information 5 [file peerj-09-12651-s005.pdf]

Specimen	Scan	Apatite Peak Position
2018-L1	pos1_darkredfill	963.3
2018-L1	pos2_transorange2	963.3
2018-L1	pos3_transfill2	963.3
2018-L1	pos3_transfill3	963.3
2018-L1	pos4_bone	961.5
2018-L1	pos4_emptyvessel	963.3
2018-L1	pos4_sphere1	959.8
2018-L1	pos4_sphere3	965
2018-L1	pos5_sphere5	963.3
2018-L1	pos6_darkredsphere	959.8
2018-L1	pos7_transareaWredspecks2	961.5
2018-L1	pos7_transareaWredspecks3	963.3
2018-L1	pos8_bone	963.3
2018-L2	pos1_transredorange	961.5
2018-L2	pos2_bone	961.5
2018-L2	pos2_fracture	961.5
2018-L2	pos2_indistinctredorange2	961.5
2018-L2	pos2_indistinctredorange3	963.3
2018-L2	pos2_redfill	965
2018-L2	pos2_round4	963.3
2018-L2	pos2_round5	961.5
2018-L2	pos5_NOorangestain	961.5
2018-L2	pos5_orangestain	961.5
2018-L3	pos1_round2	961.5
2018-L3	pos1_round3	963.3
2018-L3	pos2_sphere1	963.3
2018-L4	pos1_indistroundfill	961.5
2018-L4	pos1_roundfill2	961.5
2018-L4	pos2_roundfill	961.5
2018-L4	pos2_roundfill2	963.3
2018-L4	pos3_sphere	963.3
2018-L4	pos3_sphere3	961.5
2018-L4	pos3_sphere4	961.5
2018-L4	pos3_sphere5	963.3

Table 1. Apatite peak positions across 34 individual scans of serial section 2018-L1 – 2018-L4.

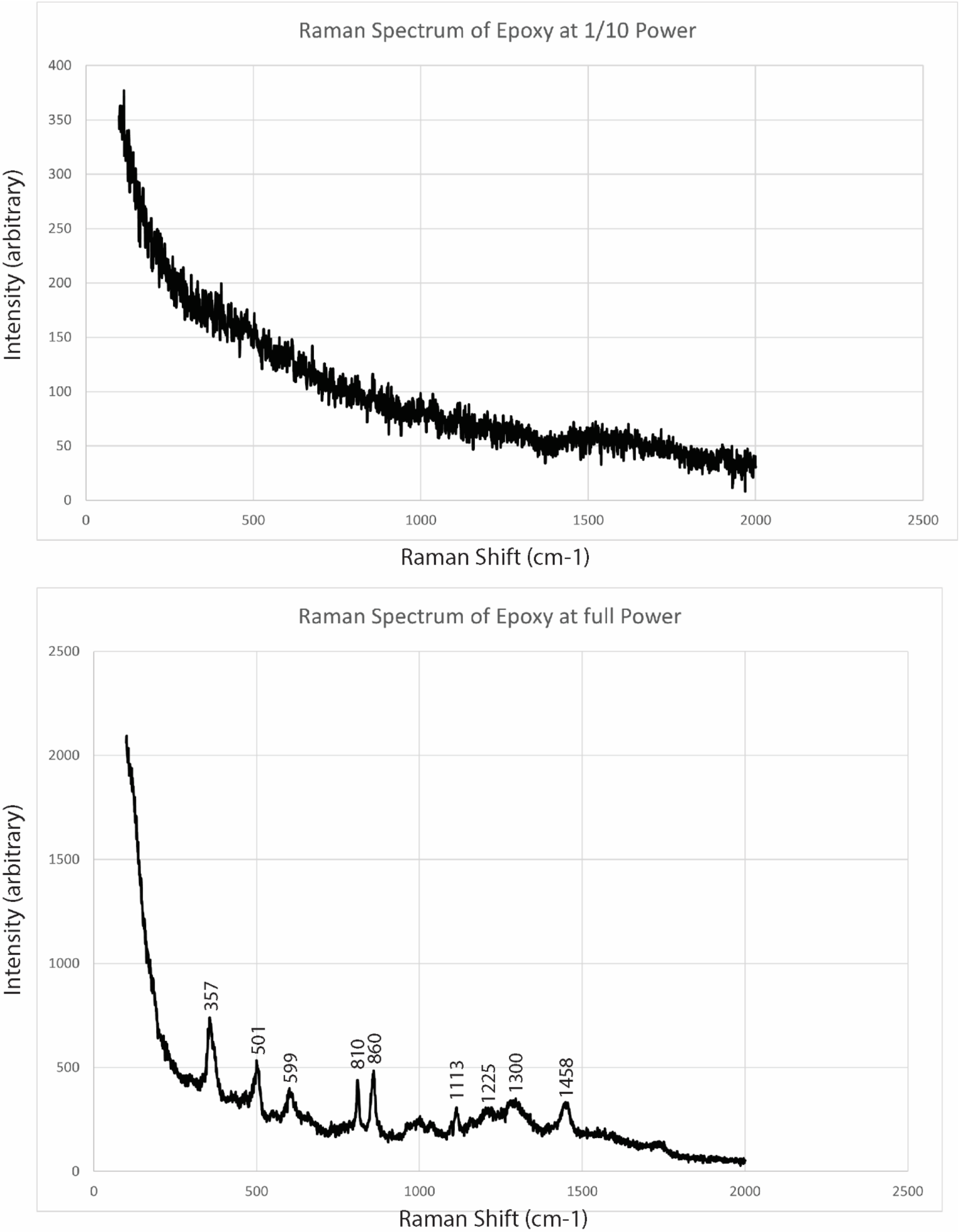

Figure 1. Raman spectra of epoxy used in this study affixed to plexiglass with superglue. These peaks are only distinguishable at a laser power not used on any of the samples in this study. They also do not line up with the sample peaks reported in the manuscript.

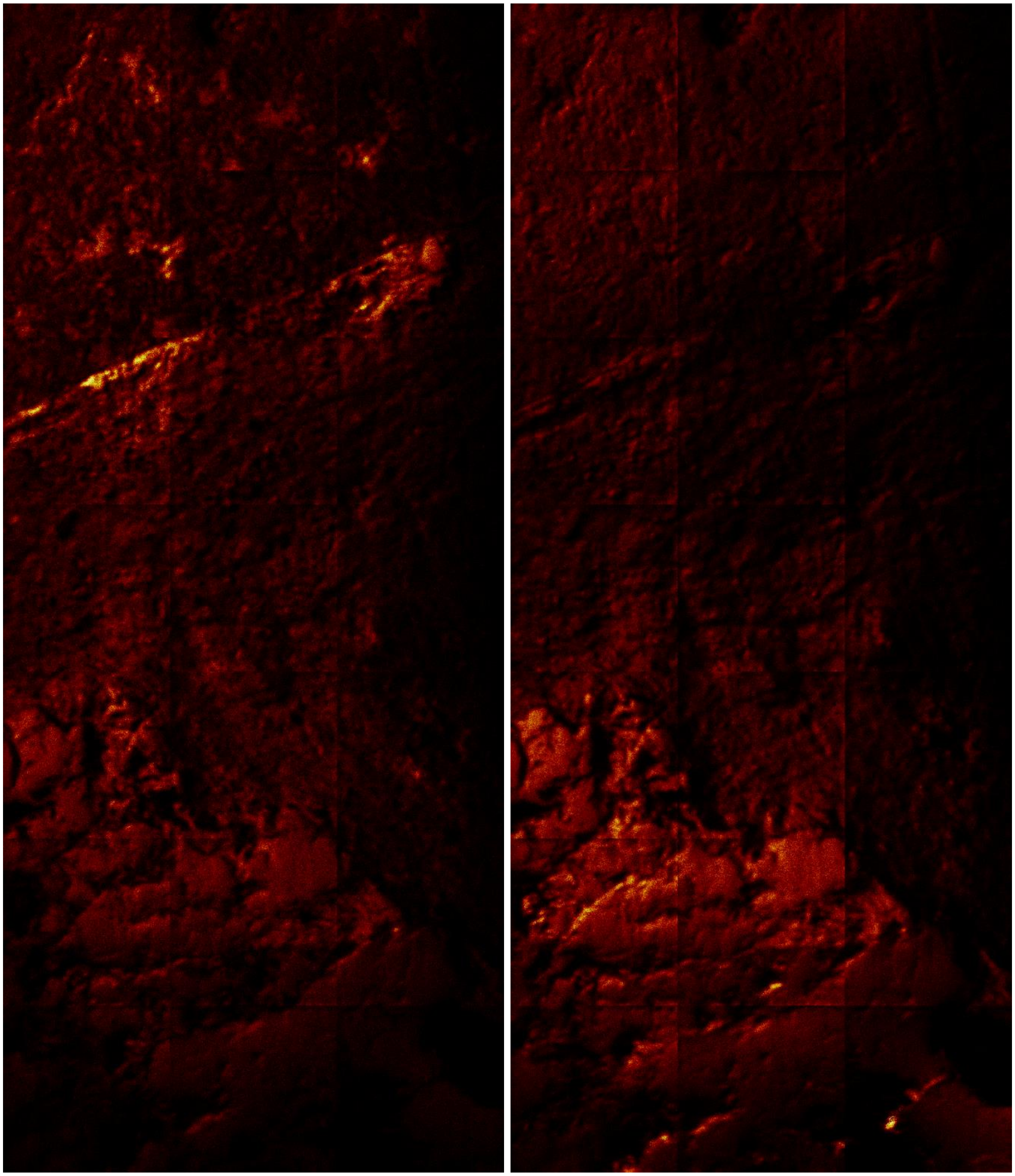

Figure 2. TOF-SIMS scans of FeO^- (left) and Mg_2O^- (right) over the same sample area described in Figure 6. See Figure 6 caption for context.
